# Amplicon sequencing analysis of arbuscular mycorrhizal fungal communities colonizing maize roots in different cover cropping and tillage systems

**DOI:** 10.1038/s41598-020-58942-3

**Published:** 2020-04-03

**Authors:** Masao Higo, Yuya Tatewaki, Karen Iida, Kana Yokota, Katsunori Isobe

**Affiliations:** Department of Agricultural Bioscience, College of Bioresource Sciences, Nihon University, Fujisawa, Kanagawa Japan

**Keywords:** Symbiosis, Microbial communities, Microbial ecology, Soil microbiology, Arbuscular mycorrhiza

## Abstract

Our understanding regarding the influence of intensive agricultural practices, including cover cropping and tillage, on communities of arbuscular mycorrhizal fungi (AMF) is lacking. This would prove to be an obstacle in the improvement of current maize (*Zea mays* L.) production. Therefore, using amplicon sequencing, we aimed to clarify how AMF communities and their diversity in maize roots vary under different cover cropping systems and two types of tillage (rotary and no tillage). Two kinds of cover crops (hairy vetch and brown mustard) and fallow treatments were established with rotary or no tillage in rotation with maize crops. Tillage and no tillage yielded a set of relatively common AMF operational taxonomic units (OTUs) in the maize crops, representing 78.3% of the total OTUs. The percentage of maize crop OTUs that were specific to only tillage and no tillage were 9.6% and 12.0%, respectively. We found that tillage system significantly altered the AMF communities in maize roots. However, the AMF communities of maize crops among cover cropping treatments did not vary considerably. Our findings indicate that compared with cover cropping, tillage may shape AMF communities in maize more strongly.

## Introduction

Growing cover crops and practicing conservation tillage are agricultural practices worldwide that have been recommended to promote soil fertility. In particular, Williams *et al*.^1^ reported that conservation tillage, including reduced or no tillage, modifies water-holding capacity and structure of soil in conservative agricultural systems. In general, conservation tillage can improve soil aggregation, increase the amount of soil organic carbon in the surface layer, and reduce erosion^2,3^. Moreover, conservation tillage enhances soil microbial diversity and the abundance of beneficial functional soil microorganisms^3,4^. The soil microbial communities stimulated by conservation tillage can play important roles in soil aggregation, soil carbon sequestration, and soil nutrition; improve water use efficiencies; and influence crop yields^5,6^.

The practice of cover cropping potentially reduces soil and wind erosion^7^, enhances soil organic matter^8^, inhibits weed establishment^9^, and increases the abundance and activity of arbuscular mycorrhizal fungi (AMF)^10^. Indeed, crops with AMF have a greater capacity to take up phosphorus (P), zinc (Zn), and water^10^ than crops without AMF. This greater capacity generally results in robust crop growth under conditions of limited nutrient availability or drought. AMF also contribute to increasing plant resistance against pathogens and help make crops generally healthier^11–13^. AMF also contribute to crop health indirectly because of their effects on soil processes in agricultural settings^14,15^. In particular, they can contribute to soil health, soil aggregate formation, and soil stability by increasing the soil’s nutrient cycling and organic matter content^16,17^. However, certain agricultural practices have negative influences on AMF abundance and functions^18,19^. For example, conventional tillage systems, including chisel plowing, rotary tillage, and disc harrowing, can disrupt the AMF hyphal network^20^, inhibit AMF development and decrease AMF abundance in soil^21^. Fallowing during the winter also decreases the AMF abundance in soil and reduces the benefit of AMF on crops^22,23^. In contrast, no-tillage or reduced tillage systems can increase the abundance and hyphal network of AMF in soil^20,24^. Growing cover crops as pre-crops can increase indigenous AMF abundance in soil and root colonization by AMF, thus improving the growth and yield of subsequent crops^10,25–27^. Furthermore, the introduction of mycorrhizal pre-crops combined with a no-tillage system increases early stage maize (*Zea mays* L.) growth and AMF colonization to a greater extent than that of mycorrhizal pre-crops combined with a rotary-tillage system^28^. Thus, combining cover crop systems with conservation tillage may provide many benefits that improve subsequent crop performance.

Moreover, there is clear evidence that AMF community structure and diversity improve plant growth performance^29^. Increased AMF richness in agricultural ecosystems has been suggested to improve crop performance^30^, while co-inoculation of AMF taxa belonging to different families improves plant biomass and mineral nutrition more efficiently than mono-inoculation^31–33^. In terms of agricultural management practices, tillage^34,35^ and cover cropping^27,36^ can alter the AMF community structure and diversity in soil and roots. For instance, Oehl and Koch^37^ reported AMF spore communities in conventional tillage are distinct from those in no-tillage systems in a vineyard farm, based on AMF spore morphology. Morimoto *et al*.^38^ showed that AMF communities in the roots of subsequent soybean crops differed, depending on their rotation with winter wheat or fallow. Other studies have shown that AMF communities can be shaped in subsequent crops, and the effect of the identity of the host crop is stronger than that of the cover crops^27,39,40^. Aside from these conflicting results, most other studies have investigated only the impact of cover cropping on AMF communities in subsequent crops^27,39,40^ and there is a lack of information about the impacts on AMF communities in subsequent crops under different agricultural management systems, such as the combination of cover cropping with tillage systems. Given our lack of knowledge regarding AMF communities in combined cover crop rotations with different tillage systems, it is imperative to understand how combining cover cropping with different tillage systems change AMF communities in subsequent crops using next-generation sequencing techniques (Illumina MiSeq Platform, etc.).

It is currently unclear which factor, cover cropping or tillage, has a greater influence in the shifts in the AMF communities in the roots of subsequent crops under cover crop rotational systems. Little is also known about how the combination of cover cropping with different types of tillage (rotary tillage and no tillage) drives shifts in the AMF communities in the roots of subsequent crops. Knowing whether such management affects the soil status and its connections to AMF taxa may help to determine the proper practices under specific agricultural settings. We hypothesized that:

1. The AMF communities of subsequent crops in cover cropping combined with rotary tillage are distinct from those in cover cropping combined with no tillage.
2. The AMF communities of subsequent crops in a rotary-tillage system will have relatively more taxa that can tolerate stress in the presence of disturbance compared to those in a cover cropping system combined with no tillage.

Illumina amplicon sequencing is a commonly used and helpful technique for AMF community analysis^41–45^. Thus, we used this technique to investigate how and whether AMF communities in the roots of maize crops change in different types of cover cropping systems combined with rotary or no tillage.

## Materials and Methods

### Experimental design of the field experiment

We performed a cover crop-maize rotational study at a Nihon University research field in Kanagawa, Japan. The research field soil is classified as an Allophonic andosol (volcanic ash soil). We combined two different cover crops [hairy vetch (*Vicia villosa* Roth., var. Fujiemon) and brown mustard (*Brassica juncea* (L.) Czern & Coss., var. Karajin)] and one bare fallow treatment with rotary or no tillage as our experimental design (Table 1). The treatments were rotated annually with maize (var. P1690). All plots had dimensions of 4 m × 5 m with three replicates, arranged according to a randomized complete block design. Regardless of tillage management, on November 10, 2016, we used a drill-seeder to plant the two cover crops at a spacing of 40 cm in rows. On April 24, 2017, we terminated both cover crops using a hammer knife mower (HRC662B, Iseki Co., Ltd., Ehime, Japan) and the aboveground parts of both cover crops remained on the soil surface in the no-tillage system. In the rotary-tillage plot, the aboveground plant parts were incorporated into the soil using a rotary tiller (KRA850, Kubota Co., Ltd., Osaka, Japan). After cover cropping or bare fallow treatment with or without rotary tillage, we planted maize at a spacing of 75 cm × 20 cm on May 18, 2017. After planting maize, each treatment received N, P_2_O_5_, and K_2_O at the rate of 200, 150, and 200 kg ha^−1^, respectively.

**Table 1:** Summary of cover crop types and tillage practices in the present experiment.

Tillage	Cover crop type	Sowing time	Termination and incorporation^b^	Summer crop type	Sowing time
Rotary tillage	Bare fallow	—	—	Maize	—
	Hairy vetch	10-Nov-16^a^	24-Apr-17	Maize	18-May-17
	Brown mustard	10-Nov-16	24-Apr-17	Maize	18-May-17
No-tillage	Bare fallow	—	—	Maize	—
	Hairy vetch	10-Nov-16	24-Apr-17	Maize	18-May-17
	Brown mustard	10-Nov-16	24-Apr-17	Maize	18-May-17

^a^DD/MM/YY.

^b^A hammer knife mower was used to terminate both cover crops at both tillage management systems. Rotary tillage: the aboveground plant parts of cover crops were incorporated into the soil by a rotary tiller. No tillage: the aboveground plant parts of cover crops remained on the soil surface.

### Soil and root collection, and root staining procedure

Soil samples in each plot comprised 10 soil cores (4 cm diameter, 0−20 cm depth) randomly collected using a core sampler (DIK-102A, Daiki Rika Co., Ltd., Saitama, Japan). On May 17, 2017, we pooled the soil samples into one composite soil sample. The roots of maize crops were collected at the stage of eight fully emerged leaves (V8 stage) on July 3, 2017. We collected maize root samples from nine plants (15-cm diameter, 0−20 cm depth) per plot. After collecting the root samples, we stored them at −80 °C for subsequent staining and DNA extraction. The roots were stained with a 3,3′-diaminobenzidine (DAB) solution^46^, and we counted AMF colonization according to Giovannetti and Mosse^47^.

### Measurement of soil biochemical properties

Soil biochemical properties were measured after cover cropping. We analyzed soil pH (soil:water ratio of 1:2.5 w/v) with a digital pH and conductivity meter (HI 9811, HANNA), and soil nitrate-nitrogen (NO_3_-N) content was measured using a LAQUA Twin nitrate meter (Horiba, Ltd., Kyoto, Japan). Extractable P (available P) from all soils, regardless of tillage management, was obtained as previously described^48^ and measured using molybdenum blue method at 710 nm using a UV-1700 Spectrophotometer (Shimadzu Co. Ltd., Japan). Soil acid phosphatase (ACP), alkaline phosphatase (ALP), and β-glucosidase activities in the soils were measured according to Ishii and Hayano^49^ and Hayano^50^.

### Processing of samples for amplicon sequencing

We froze 100 mg of fresh maize roots from all plots and retrieved total genomic DNA from the roots using the DNA suisui-P kit (RIZO, Tsukuba, Japan)^27^ following the manufacturer’s instructions. DNA solution was stored at −30 °C until use. For nested PCR, we targeted a partial sequence of the small subunit of the ribosomal RNA gene (SSU rDNA) region using a previously described PCR method^27^. We selected the primer pair AM1^51^/NS31^52^ for the first PCR to amplify a partial region of the 18S rDNA of AMF taxa. The first PCR was performed in 10-μl reactions, each consisting of 2 × Platinum™ Green Master Mix (Thermo Fisher Scientific Inc), 1 µl of template DNA, and 0.4 μM of AM1/NS31 primers. For the second PCR procedure, we diluted the amplicons from the first PCR 10-fold and used these amplicons as a template for the AMV4.5NF/AMDGR^53^ primers that are attached via the Illumina MiSeq adapter sequences. The second PCR was performed in 10 μl reactions comprising 1 µl of template DNA, 2 × Platinum™ Green Master Mix, and 0.3 μM of AMV4.5NF/AMDGR primers. Then we added both the Illumina MiSeq adapter sequences and an 8-bp barcode sequence as an index to distinguish each PCR amplicon.

### Miseq amplicon sequencing of AMF communities in roots

The Illumina MiSeq amplicons were sequenced to determine AMF communities in the roots of maize crops following procedures reported by Higo *et al*.^27,54^. Briefly, we washed and cleaned the amplicons from the second PCR before amplicon sequencing. These products were paired-end (PE) sequenced (2 × 300 bp) using an instrument of Illumina MiSeq amplicon sequencing at the Bioengineering Lab Co., Ltd. We performed the processing of sequence reads using QIIME version 1.9.1^55^. The PE reads were truncated at any site that received an average quality score of <20 over a 40-bp sliding window, and truncated reads shorter than 40 bp were discarded using the FASTX-Toolkit (http://hannonlab.cshl.edu/fastx_toolkit/index.html). We assembled the PE reads through their overlapping sequences that had a minimum overlap length of 10 bp and we deleted the reads that could not be assembled. We used Fast length adjustment of short reads (FLASH) ver. 1.2.11 to assess the clean sequences. Chimeric sequences were removed using UCHIME in USEARCH ver. 10.0.240. We then utilized the BLAST function in the Maarj*AM* database (https://maarjam.botany.ut.ee/) to determine and group the operational taxonomic units (OTUs) at 97% similarity. Additional taxonomic assignment was based on phylogenetic relationships. Representative sequences were aligned with known AMF taxa from NCBI Genbank and a neighbor-joining phylogenetic tree with 1000 bootstrap replicates was created using MEGA 7^56^ (see Supplementary Fig. S1). The raw data sequences have been deposited at the Sequence Read Archive of the DNA Data Bank of Japan (DDBJ) under Bio Project Accession number PRJDB7275.

### Statistical analysis

Root staining data reflecting AMF colonization was transformed into the arcsine-square root values for normalization. The differences in the mean values of each parameter among tillage and cover cropping systems were evaluated by Tukey’s test and then two-way analysis of variance (ANOVA) was conducted using the *emmeans*^57^ package in R 3.6.1 (www.r-project.org). Before molecular bioinformatic analysis, we resampled according to the lowest number of reads to equalize the assessment among all treatments regardless of Illumina amplicon read depth. After rarefaction analysis, we calculated the Hill number 0D as OTU richness (q = 0), the Shannon index (the exponential of Shannon entropy, q = 1), and the Simpson index (the inverse Simpson concentration, q = 2) from data on the diversity of AMF communities using the “*renyi*” function^58^ of the *vegan* package^59^. Additionally, to test whether AMF OTUs were significantly related to rotary tillage and no tillage with cover cropping, we performed a species indicator analysis. We also estimated values of indicator species using “*multipatt*” function^60^ in the *indicspecies* package to test for statistical significance of the highest OTU association in each plot.

To demonstrate the variations in the AMF communities in maize crops among tillage management and cover cropping, we performed distance-based redundancy analysis (db-RDA) in the *vegan* package in R.3.6.1^59^. Goodness-of-fit (*R*^2^) for measured factors fitted to the db-RDA ordination of the AMF communities were calculated using the “*envfit*” function in the *vegan* package with *P*-values based on 999 permutations^59^. A permutational multivariate analysis of variance (PERMANOVA) was performed with 9999 permutations using the “*adonis*”^59^ function in the *vegan* package to investigate if AMF communities differed significantly in tillage management and cover cropping. In addition, a multinomial species classification method^27,61^ using the package *vegan* were introduced to recognize “specialist OTUs” and “generalist OTUs” for tillage or no tillage.

## Results

### Soil biochemical properties and AMF colonization of maize roots among different cover cropping practices with rotary or no tillage

**Figure 1:**
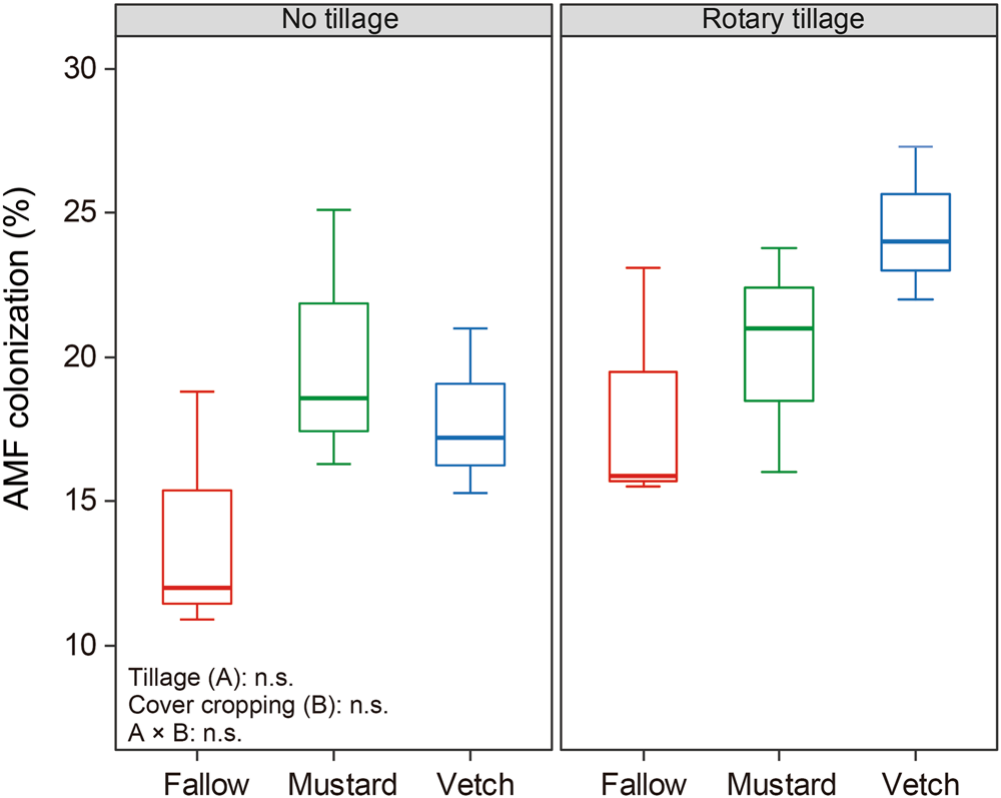
Influence of cover crops and tillage management on the colonization of arbuscular mycorrhizal fungi (AMF) in maize roots at the eight fully emerged leaves stage. Horizontal bold lines explain the median number; vertical lines explain minimum and maximum numbers of the plots and box margins ± standard errors. n.s. indicates no significant difference by two-way analysis of variance. All analyses and Fig. 1 were run and created in the software environment RStudio (Version 1.2.1335 – © 2009–2019 RStudio, Inc.) (http://www.rstudio.com).

Two-way ANOVA shows that the NO_3_-N content in soil was significantly influenced by cover cropping, but soil pH was not affected by either tillage or cover crop management (Table 2). The two-way ANOVA allows interpretation of main effects and interaction, therefore, we performed the Tukey test only in the measured variables that there was a significant difference by cover crop management. In particular, the average NO_3_-N content for hairy vetch was significantly higher than those in other cover crops. The available soil P was higher in all rotary tilled treatments than in no tilled ones. Conversely, the activity of ACP, ALP, and β-glucosidase in soil was significantly affected by cover cropping. The activity of average ACP and ALP for hairy vetch and brown mustard was significantly higher than that in fallow. The activity of average β-glucosidase showed a similar trend, but a significant effect of tillage was observed in hairy vetch, where the activity raised in no tilled treatment, in opposition to fallow and brown mustard, where remained unchanged. A significant difference in the colonization by AMF among other cover crop treatments regardless of tillage management was not detected (Fig. 1).

**Table 2:** Influence of cover crops and tillage management on the soil biochemical properties before planting maize.

Plots	pH	NO_3_-N		Available P	ACP activity		ALP activity		β-glucosidase activity	
	(H_2_O)	(mg/kg)		(mg P/kg)	(mU/g)		(mU/g)		(mU/g)	
RT + Fallow^a^	5.4 ± 0.03^b^	30.5 ± 2.0		67.7 ± 22.4	13.9 ± 0.8		29.5 ± 0.5		3.7 ± 0.1	c^d^
RT + Brown mustard	5.3 ± 0.09	51.0 ± 6.5		53.3 ± 10.1	20.7 ± 0.6		32.5 ± 1.9		8.2 ± 0.7	bc
RT + Hairy vetch	5.2 ± 0.06	67.8 ± 11.3		52.0 ± 19.5	23.7 ± 2.4		36.0 ± 1.2		11.3 ± 1.1	b
NT + Fallow	5.4 ± 0.09	35.4 ± 3.3		15.4 ± 3.1	14.2 ± 0.4		28.4 ± 1.4		3.2 ± 0.1	c
NT + Brown mustard	5.2 ± 0.03	43.1 ± 12.3		20.3 ± 4.3	19.1 ± 1.3		37.1 ± 1.7		7.4 ± 1.0	bc
NT + Hairy vetch	5.4 ± 0.11	64.0 ± 1.9		36.1 ± 4.5	19.4 ± 1.2		38.0 ± 1.9		18.9 ± 3.5	a
Rotary tillage	5.3 ± 0.04	59.8 ± 13.5		57.7 ± 9.4	19.4 ± 1.6		32.7 ± 1.2		7.7 ± 1.2	
No tillage	5.3 ± 0.05	47.5 ± 5.7		24.0 ± 3.7	17.6 ± 1.0		34.5 ± 1.7		9.8 ± 2.6	
Fallow	5.4 ± 0.05	33.0 ± 2.1	B^c^	41.6 ± 15.5	14.1 ± 0.4	B	29.0 ± 0.7	B	3.4 ± 0.1	B
Brown mustard	5.3 ± 0.04	47.1 ± 6.5	B	36.8 ± 8.9	19.9 ± 0.7	A	34.8 ± 1.6	A	7.8 ± 0.6	B
Hairy vetch	5.3 ± 0.08	65.0 ± 2.7	A	44.1 ± 9.6	21.5 ± 1.6	A	37.0 ± 1.1	A	15.1 ± 2.4	A
**Two-way ANOVA**^**e**^										
Tillage (A)	n.s.	n.s.		*P* < 0.01	n.s.		n.s.		n.s.	
Cover cropping (B)	n.s.	*P* < 0.01		n.s.	*P* < 0.001		*P* < 0.001		*P* < 0.001	
A × B	n.s.	n.s.		n.s.	n.s.		n.s.		*P* < 0.05	

^a^RT: rotary tillage. NT: no tillage.

^b^Means ± standard errors.

^c^Different capital letters within the same column for NO_3_-N content, activities of acid, alkali phosphatase and β-glucosidase among mean values of three cover crop types indicate significant differences at the 5% level by the Tukey test.

^d^Different lower letters within the same column for β-glucosidase activity among each treatment indicate significant differences at the 5% level by the Tukey test.

^e^A significant difference at the 5%, 1% and 0.1% levels by cover cropping and tillage practices were explained by two-way analysis of variance. All analyses were run in the software environment RStudio (Version 1.2.1335 – © 2009–2019 RStudio, Inc.) (http://www.rstudio.com).

### Maize root AMF community taxa after cover cropping with tillage systems

**Figure 2:**
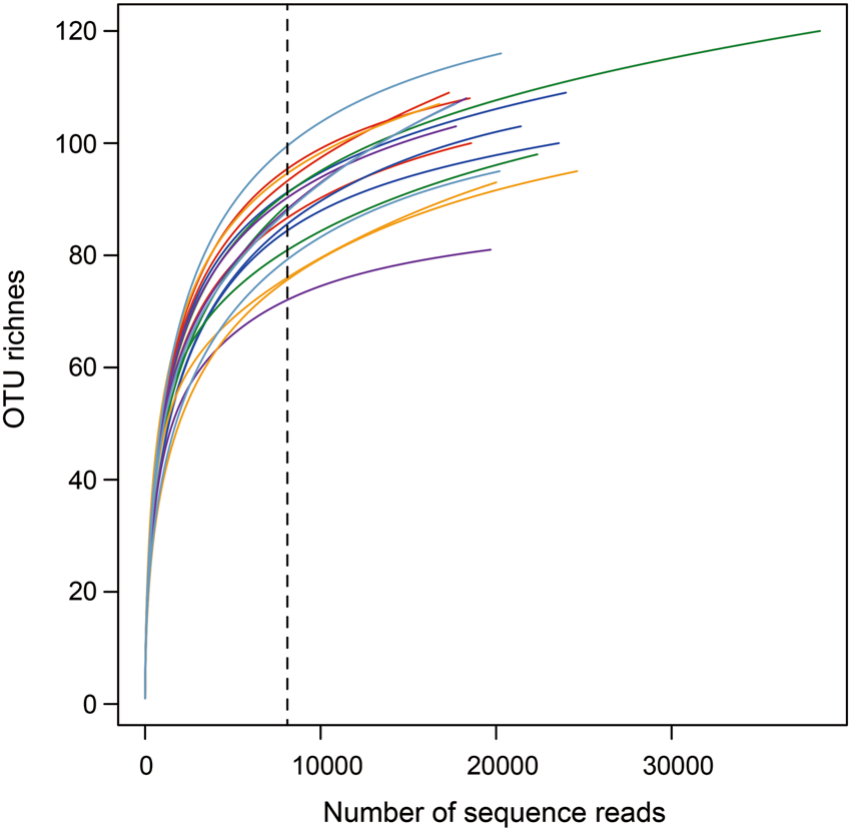
Depths of Illumina amplicon sequencing in the maize roots by rarefaction analysis. The vertical dashed line was placed at 8,104 sequences and the operational taxonomic units (OTUs) of arbuscular mycorrhizal fungi (AMF) were defined at a cut-off level of 8,104 reads. All analyses and Fig. 2 were run and created in the software environment RStudio (Version 1.2.1335 – © 2009–2019 RStudio, Inc.) (http://www.rstudio.com).

**Figure 3:**
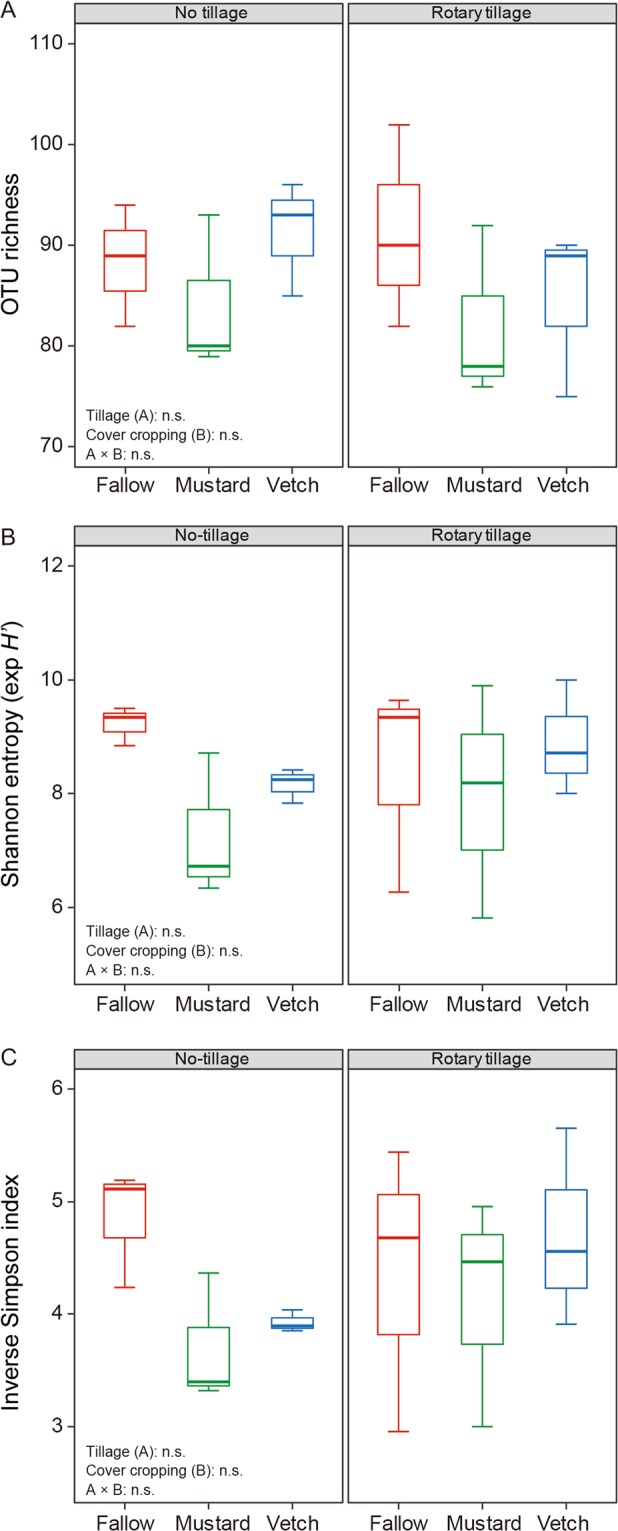
Influence of cover crops and tillage management on the Hill numbers in the maize roots at the eight fully emerged leaves stage. (**A**) The Hill number 0D as operational taxonomic unit (OTU) richness, (**B**) Shannon index (the exponential of Shannon entropy), and (**C**) Simpson index (the inverse Simpson concentration). n.s. means no significant differences by two-way analysis of variance. Horizontal bold lines explain the median number; vertical lines explain minimum and maximum numbers in the plots and box margins ± standard errors. n.s. indicates no significant difference by two-way analysis of variance. All analyses and Fig. 3 were run and created in the software environment RStudio (Version 1.2.1335 – © 2009–2019 RStudio, Inc.) (http://www.rstudio.com).

**Figure 4:**
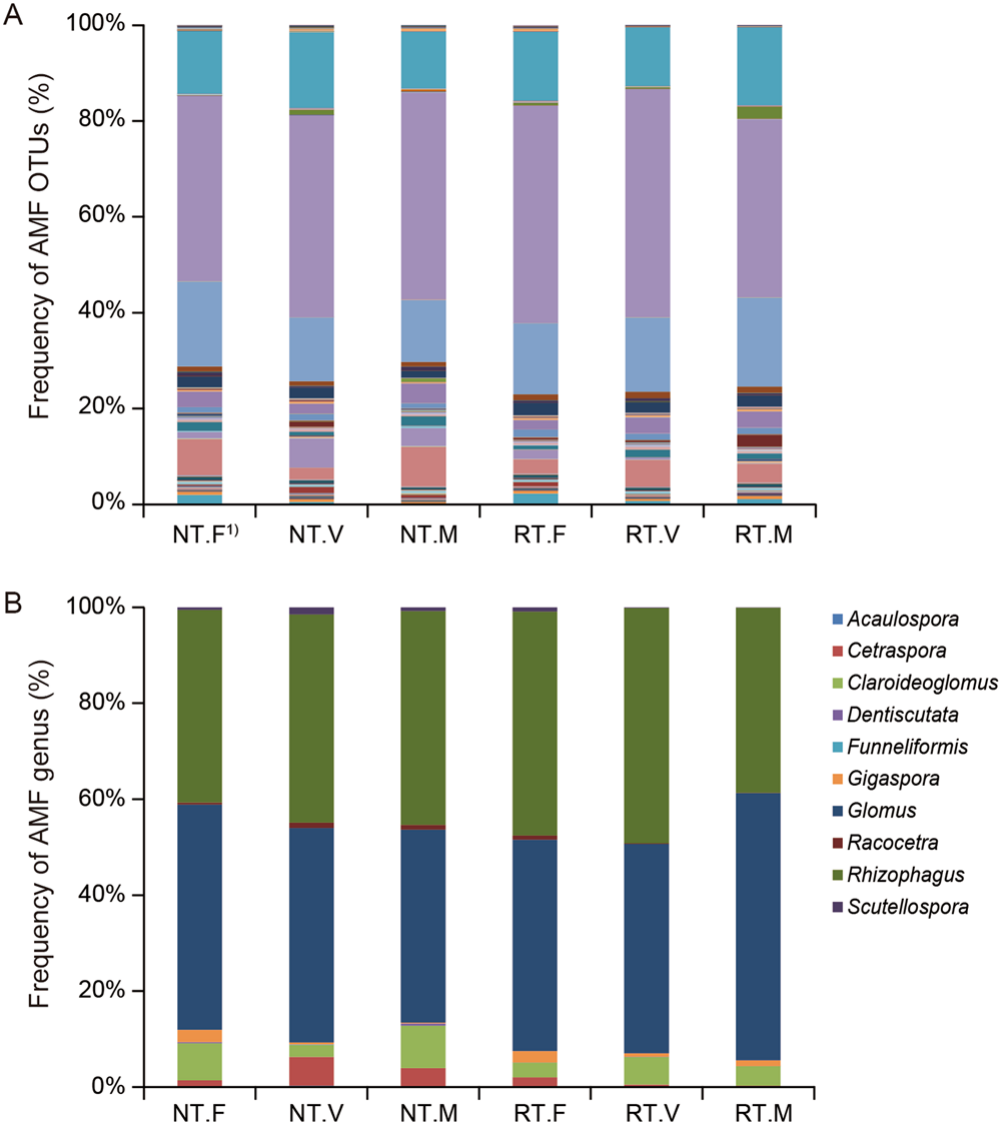
Relative abundance of operational taxonomic units (OTUs) and genus of arbuscular mycorrhizal fungi (AMF) in the maize roots at the eight fully emerged leaves stage. 1) RT: rotary tillage, NT: no tillage. F: fallow, M: brown mustard, V: hairy vetch. A = OTU-based abundance, B = genus-based abundance.

We detected a total of 148 AMF OTUs in the roots of maize (Fig. 2 and Supplementary Table S1). Regardless of tillage management, the Hill numbers did not differ significantly, including AMF OTU richness, Shannon index (the exponential of Shannon entropy), and Simpson index (the inverse Simpson concentration) among cover cropping systems (Fig. 3A–C). Moreover, the relative abundance of AMF OTUs did not differ significantly among cover cropping treatments with or without rotary tillage (Fig. 4A and Supplementary Table S2A). Overall, *Glomus* and *Rhizophagus* genera were the most abundant in maize roots (average of 45.9% and 43.8%, respectively) (Fig. 4B and Supplementary Table S2B). The relative abundance levels of *Claroideoglomus* and *Cetraspora* genera were 5.3%, and 2.3%, respectively. We also found relatively low abundance levels of *Gigaspora*, *Racocetra*, *Scutellospora*, *Acaulospora*, *Dentiscutata*, and *Funneliformis* in the roots of maize crops (1.2%, 0.6%, 0.6%, 0.1%, 0.1%, and 0.02%, respectively).

**Figure 5:**
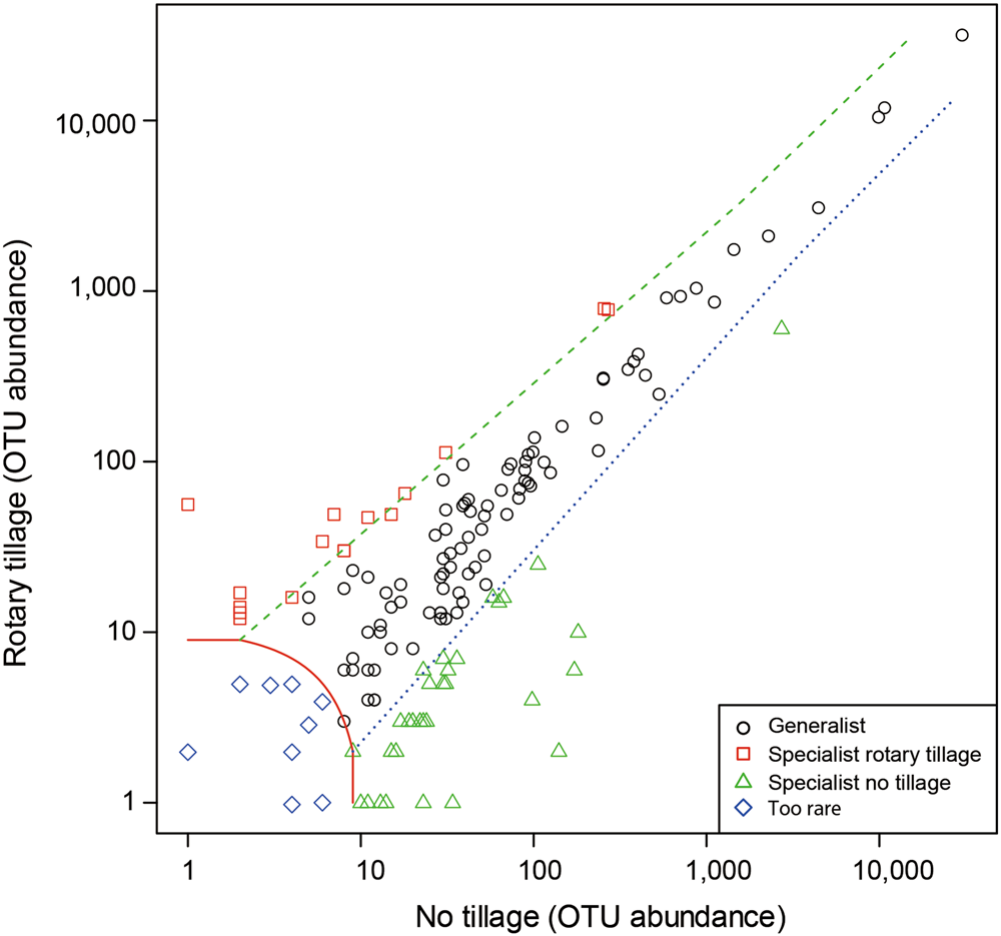
Multinomial species classification of taxa in the maize roots grown under rotary tillage and no tillage. The operational taxonomic units (OTUs) of arbuscular mycorrhizal fungi (AMF) common to both rotary tillage and no tillage are represented by circles; those observed only in rotary tillage are represented by squares, and those observed only in no tillage are represented by triangles. Diamonds represent rare AMF OTUs. All analyses and Fig. 5 were run and created in the software environment RStudio (Version 1.2.1335 – © 2009–2019 RStudio, Inc.) (http://www.rstudio.com).

Generalist taxa were represented by 66.7% of the OTUs that were commonly shared in the roots of maize crops undergoing both tillage and no-tillage treatments (Fig. 5 and Supplementary Table S3). In total, 10.7% and 18.7% of the AMF OTUs were unique to only rotary tillage or no tillage, respectively. Our assessment of the values of indicator species suggests that six AMF taxa are indicators that were significantly affected by tillage management; these mainly include *Glomus* and uncultured Glomeromycotina taxa in the rotary-tillage system (Supplementary Table S4).

### Differences in AMF OTU communities of maize roots

**Figure 6:**
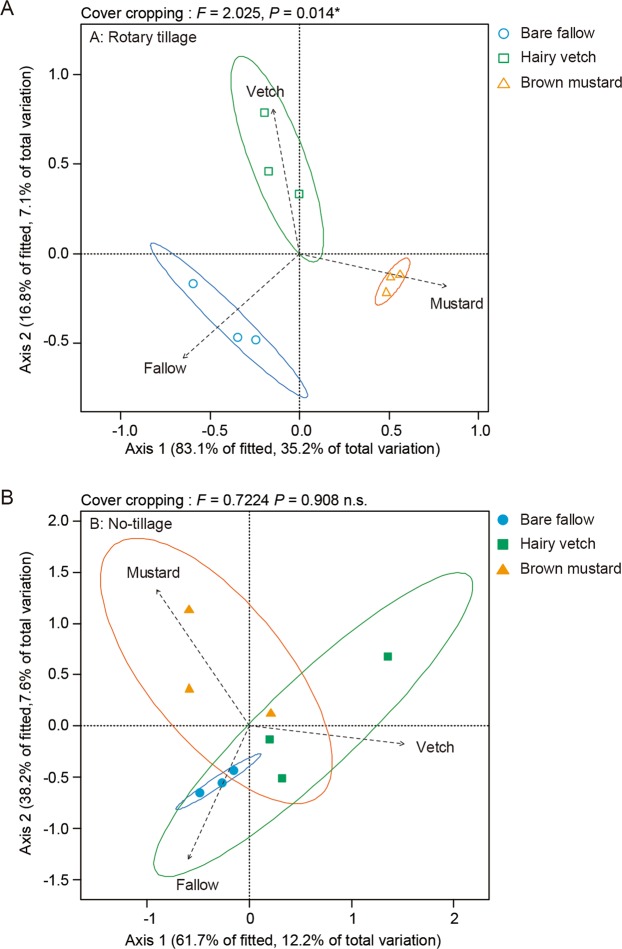
Distance-based redundancy analysis (db-RDA) of the influence of cover crop type in each tillage management system on the communities of arbuscular mycorrhizal fungi (AMF) in the maize roots. Dashed lines explain the effect of each type of cover crop. Ellipses represent 95% confidence intervals. A: rotary tillage, B: no tillage. All analyses and Fig. 6 were run and created in the software environment RStudio (Version 1.2.1335 – © 2009–2019 RStudio, Inc.) (http://www.rstudio.com).

Among rotary-tillage treatments, bare fallow (Monte-Carlo permutation test: *R*^2^ = 0.681, *P*-value = 0.024) and brown mustard (*R*^2^ = 0.837, *P*-value = 0.013) significantly affected the AMF communities (Fig. 6A), while hairy vetch (*R*^2^ = 0.395, *P*-value = 0.233) did not affect the AMF communities of maize crops. Among the no-tillage treatments, only brown mustard (*R*^2^ = 0.650, *P*-value = 0.031) significantly affected the AMF communities (Fig. 6B). Among the rotary-tillage treatments, PERMANOVA reveals that the cover crop treatment significantly affected the AMF communities of maize crops (PERMANOVA: *F*-statisti*c* = 2.025, *P*-value = 0.014). However, cover crops did not significantly affect the AMF communities in no-tillage treatments (*F*-statisti*c* = 0.738, *P*-value = 0.902).

**Figure 7:**
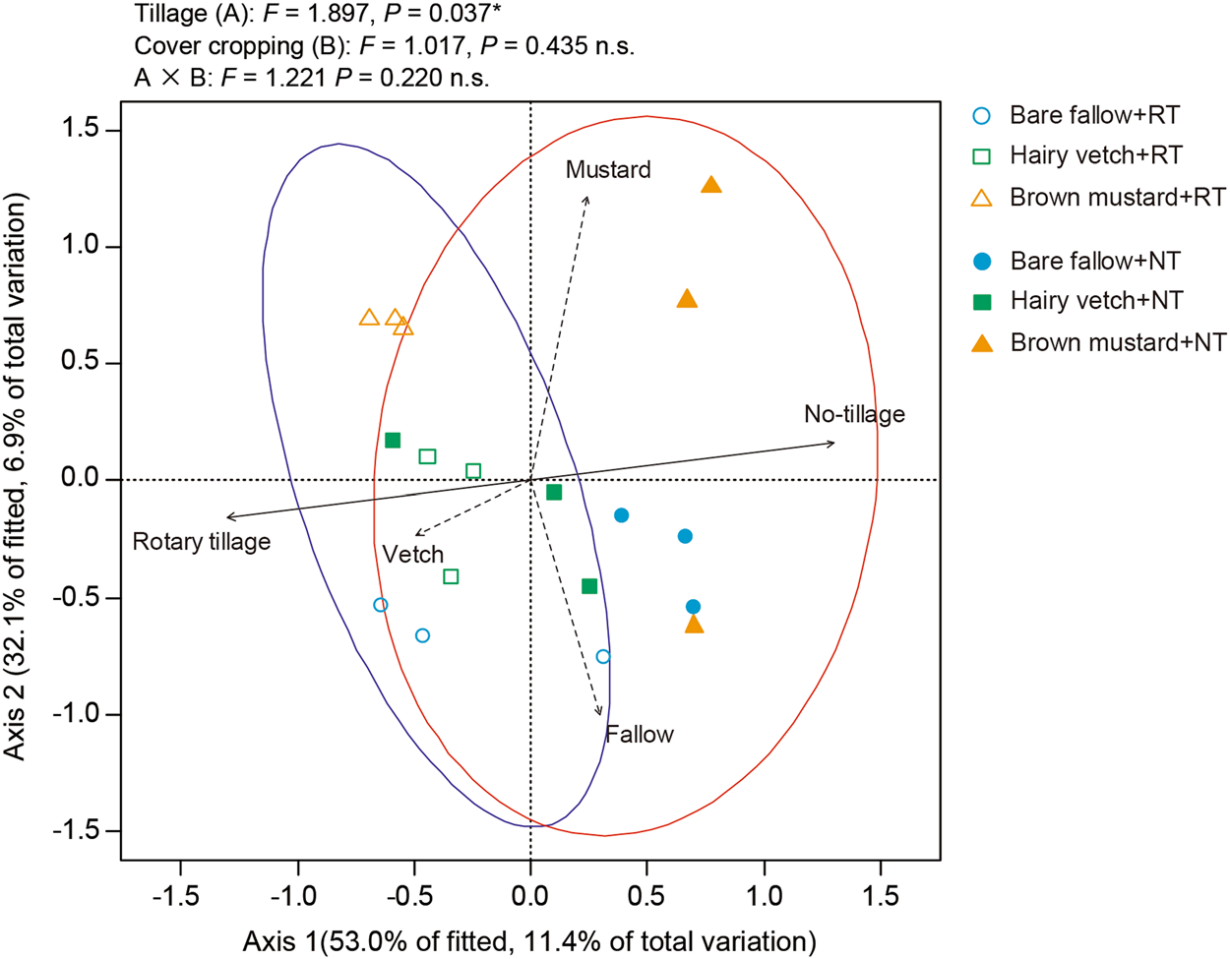
Influence of cover crop type and tillage management on the communities of arbuscular mycorrhizal fungi (AMF) in the maize roots according to distance-based redundancy analysis (db-RDA). Dashed lines explain the effect of each type of cover crop. RT: rotary tillage. NT: no tillage. Ellipses represent 95% confidence intervals. All analyses and Fig. 7 were run and created in the software environment RStudio (Version 1.2.1335 – © 2009–2019 RStudio, Inc.) (http://www.rstudio.com).

The db-RDA also found that tillage management alters the AMF communities of maize crops, while only brown mustard pre-crop significantly affected the AMF communities (Monte-Carlo permutation test: *R*^2^ = 0.507, *P*-value = 0.002) (Fig. 7). Both rotary tillage (*R*^2^ = 0.515, *P*-value = 0.007) and no tillage (*R*^2^ = 0. 515, *P*-value = 0.007) had significant effects on the AMF communities in maize crops. Moreover, PERMANOVA shows that tillage management rather than cover cropping or the interaction between tillage management and cover crop type significantly affected the AMF communities in maize crops (PERMANOVA: *F*-statisti*c* = 1.897, *P*-value = 0.037).

## Discussion

### AMF communities in maize roots

Our findings indicate that representatives of Glomeraceae (*Glomus* and *Rhizophagus*) and Claroideoglomeraceae (*Claroideoglomus*) are the main genera in maize roots (Fig. 4B and Supplementary Table S1). Current reports based on Illumina amplicon sequencing have shown that Glomeraceae taxa are generally dominant under agricultural field conditions^41–45,54^ because of their abilities to sporulate for rapid recovery and to adapt to disturbed environments^62^. Moreover, Glomeraceae are able to colonize through fragments of mycelium or through root fragments containing mycorrhizae^63^. Their hyphae are able to easily anastomose owing to their ability to re-establish a network after a mechanical disruption^64^. In contrast, members of Gigasporaceae, such as *Cetraspora*, *Gigaspora*, *Scutellospora*, *Racocetra*, and *Dentiscutata*, propagate through spore dispersal or through intact hyphae^65–67^. These factors taken together explain why Glomeraceae is generally dominant in cultivated lands, that is, it is well-adapted to agricultural settings. Additionally, PCR-based AMF community findings may also be affected by PCR bias, which is dependent on different combinations of the primer pair with the target region of the rRNA gene that are due to potentially different degrees of specificity and amplification efficiency^68^. For example, Kohout *et al*.^68^ reported that primer pairs that target the SSU and large subunit (LSU) rDNA of AMF are strongly biased toward Glomeraceae. Because our results show that Glomeraceae are dominant in maize roots regardless of tillage and cover cropping, further studies should carefully select primer pairs that avoid potential PCR and primer bias as well as technical sequencing errors.

In addition, environmental factors may shape the AMF communities^69^. For example, Gottshall *et al*.^69^ suggested that Gigasporaceae tends to be associated with soil disturbance, but the association is not statistically significant. Conversely, *R. irregularis* of the Glomeraceae, which is recognized as a generalist, has been discovered in different land-use types and can tolerate different agricultural practices, including tillage due to its ability to rapidly reconstruct its hyphal network^70^. However, intensive agricultural management practices may both simplify and shape the AMF communities according to AMF taxa preferences^35,69^. These findings indicate that the frequencies of AMF specialists and generalists within the AMF communities may change according to tillage intensity^18^. The shifts that we observed in the AMF OTUs (Figs. 4 and 5 and Supplementary Tables S2–S4) may be connected to the preference of AMF taxa in the roots of maize crops for tillage or cover cropping practices.

### Influence of tillage and cover crops on shifts in AMF communities

The beneficial impacts of cover cropping may differ among the AMF communities, and the question of whether cover crop type changes the AMF communities in the roots of maize crops remains to be investigated. Higo *et al*.^71^ reported that the pre-crop type influences maize root AMF communities, which agrees with part of the present study’s findings (Fig. 6). However, our findings suggest that cover cropping may not induce differences in the AMF communities in the subsequent maize crops as strongly tillage management (Fig. 7). This also agrees with data of Higo *et al*.^72^, who reported that rotation year was a stronger driver in the formation of root AMF communities in subsequent soybean crops during a 5-year cover cropping system. This indicates that climate conditions and/or some other environmental driver(s) may be the key in shaping AMF communities in roots.

In addition, AMF communities in maize crops varied distinctly, indicating that tillage practice altered the AMF communities in maize crops (Fig. 6), in partial agreement with previous data^73,74^. Moreover, tillage management induced more changes to the AMF communities in maize roots than the type of cover crops. However, the questions of how and whether the level of tillage intensity alters the AMF communities in agricultural settings remain unanswered. Future studies on the interactions between the AMF communities and agricultural settings should focus on how tillage intensity affects the AMF communities in maize crops rotated with cover crops.

### Environmental influences on shifts in AMF communities

Tillage and cover crop residue management are particularly imperative for controlling plant-microbe interactions and crop performance^75,76^. In general, cover crop residues that have been incorporated into the soil surface by conventional tillage systems release allelopathic substances that can inhibit weed germination, establishment, and growth^77^. In contrast, in conservation tillage systems (including no tillage and reduced tillage), the nutrients from surface-broadcasted fertilizers and surface-applied crop residues accumulate and concentrate in the topsoil, where their movements are reduced^78^. In no-tillage systems, however, crop residue decomposition rates and nutrient release into the soil are slow compared to conventional tillage systems, including rotary tillage and chisel plow^79^, which may influence the soil microbial community structures^80^. These combined findings indicate that the differences between the effects of rotary and no-tillage management systems on the AMF communities in the roots of maize crops can be explained by cover crop residues releasing allelopathic substances and nutrients due to enhanced decomposition rates (Fig. 7).

Moreover, tillage can affect soil temperature by changing soil surface conditions^81^. Crop residues remaining on the soil surface in conservation tillage systems, including no tillage and reduced tillage, can decrease the rate of soil temperature fluctuations because the surface residues reflect incident solar radiation more than the bare soil^82^. A previous study shows that soil temperature changes both the structure and allocation of the AMF hyphal network; this effect is consistent in cooled soils (soil temperature 14 °C), ambient soils (20 °C), and warmed soils (26 °C)^83^. Heinemeyer *et al*.^84^ reported that shading and soil temperature changed AMF communities in roots, with some AMF taxa being replaced (although inconsistently) by others and some disappearing throughout winter, summer and autumn. Higo *et al*.^85^ also reported on seasonal variation, finding a distinct difference between winter and spring AMF communities in the roots of winter cover crops. However, we did not investigate the effect of tillage systems and cover crop residues on soil temperature fluctuations. Future research should investigate how and whether AMF communities respond to environmental changes due to cover cropping and tillage management systems.

## Conclusions

Tillage management may be more important than cover cropping in shaping the AMF communities in maize crops. In addition, various kinds of cover cropping systems may produce various types of effects on the AMF communities in maize crop roots. The synergistic effects of tillage and cover cropping are partially responsible for shaping the AMF communities. Knowledge in this connection should be valuable in future experiments. This is because variations in the AMF communities in roots may influence crop growth performance in cropping systems with tillage management. However, we still need to clarify how the combination of tillage intensity and cover crop type is associated with individual AMF taxa in agricultural settings. Such an investigation will provide beneficial information on the appropriate cover crop selection and tillage practice for improving the functions of AMF taxa in cropping systems.

## Supplementary information

Supplementary information 1.

Supplementary information 2.
