# Supplementary material for: Amplicon sequencing analysis of arbuscular mycorrhizal fungal communities colonizing maize roots in different cover cropping and tillage systems
Source: Sci Rep. 2020 Apr 3;10:6039. doi: 10.1038/s41598-020-58942-3 (PMC7125109; doi:10.1038/s41598-020-58942-3)
Supplement: Supplementary file 1 — Supplementary information 1. [file 41598_2020_58942_MOESM1_ESM.pdf]

Supplementary information

Title: Amplicon sequencing analysis of arbuscular mycorrhizal fungal communities colonizing maize roots in different cover cropping and tillage systems

Masao Higo*†, Yuya Tatewaki†, Katsunori Isobe

Department of Agricultural Bioscience, College of Bioresource Sciences, Nihon University, Fujisawa, Kanagawa, Japan

† These authors contributed equally to this work.

*Corresponding author

Correspondence to Masao Higo

Tel: +81-466-84-3502

E-mail: higo.masao@nihon-u.ac.jp

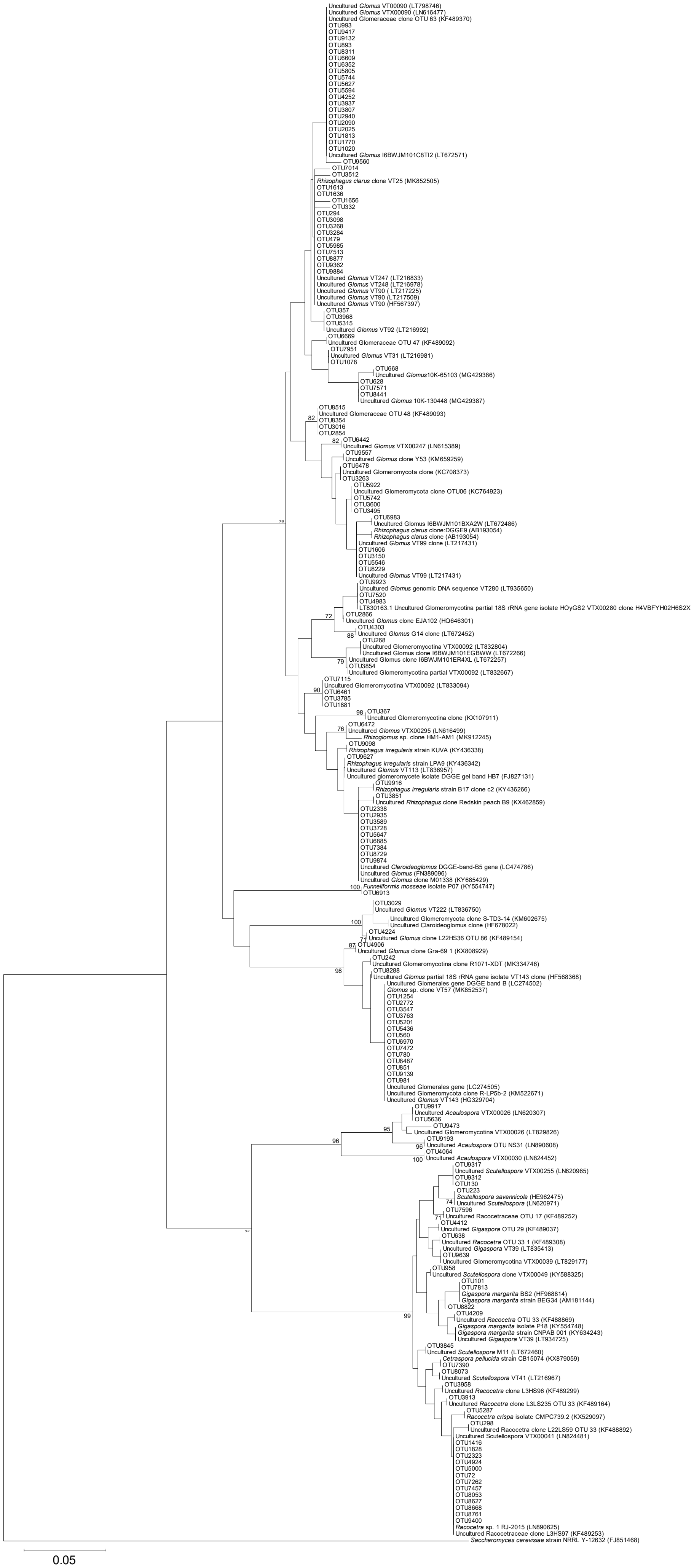

Supplementary Figure S1: A neighbor-joining tree of partial SSU rDNA sequences obtained from the roots of maize, rooted tree by *Scutellospora savannicola* as an outgroup. Bootstrap values (only values > 70 are shown) were estimated from 1,000 replicates. Representative sequences in each AMF OTU from roots are incorporated.
